# Changes in Molecular Characteristics of *Mycoplasma pneumoniae* in Clinical Specimens from Children in Beijing between 2003 and 2015

**DOI:** 10.1371/journal.pone.0170253

**Published:** 2017-01-20

**Authors:** Hongmei Sun, Guanhua Xue, Chao Yan, Shaoli Li, Hanqing Zhao, Yanling Feng, Liqiong Wang

**Affiliations:** Department of Bacteriology, Capital Institute of Pediatrics, Chaoyang District, Beijing, China; Universidad Nacional de la Plata, ARGENTINA

## Abstract

The molecular characteristics of 480 *Mycoplasma pneumoniae* polymerase chain reaction-positive specimens (331 were previously reported and 149 were newly reported) collected from pediatric patients in Beijing, China, between 2003 and 2015 were analyzed. Genotype M4-5-7-2/P1 were the most prevalent across the 13-year study period, although the isolation and mutation rates for this genotype varied between the periods 2003–2007, 2008–2013, and 2014–2015. In addition, there was a close association between the M4-5-7-2 genotype and macrolide resistance.

## Introduction

*Mycoplasma pneumoniae* is a common bacterial pathogen that causes upper and lower respiratory tract infections in humans, particularly children and young adults [1]. Worldwide epidemics of *M*. *pneumoniae* infection occur every 3–7 years [2]., and after the last epidemic around 2012, there were significant increases in the number of *M*. *pneumoniae*-associated pneumonia cases reported in 2015 in China, Japan, England, and Wales [3,4,5]., there may be another new epidemic started in 2015. Analysis of the molecular characteristics of specimens collected over this period is therefore very important.

The most prevalent *M*. *pneumoniae* genotypes, along with drug resistance profiles, can differ between regions and countries [6, 7]., and even within the same region, type-shifts in predominant P1 genotypes have been recorded in Japan at intervals of about 10 years [8]. However, there is little epidemiological information regarding trends in P1 and multiple locus variable-number tandem repeat analysis (MLVA) genotypes and macrolide resistance mutations in the 23S rRNA gene of *M*. *pneumoniae* in China. Therefore, this study aimed to elucidate changes in the molecular characteristics of *M*. *pneumoniae* specimens in China over a 13-year period.

## Materials and Methods

### Ethics statement

This study was performed in compliance with the Helsinki Declaration (Ethical Principles for Medical Research Involving Human Subjects), and was approved by the research board of the Ethics Committee of the Capital Institute of Pediatrics, Beijing, China. As all patient information was anonymized, informed consent was not needed for this study, as per the guidelines of the Ethics Committee of the Capital Institute of Pediatrics.

### Identification of *M*. *pneumoniae*-positive specimens

We analyzed the molecular characteristics of *M*. *pneumoniae* specimens directly from 480 polymerase chain reaction (PCR)-positive respiratory specimens collected at the Affiliated Children’s Hospital of the Capital Institute of Pediatrics, Beijing, China, between 2003 and 2015. Specimens collected from 2008–2013 (69.0%, 331/480) have been previously reported [6, 7, 9, 10], while specimens collected from 2003–2007(4.8%, 23/480) and 2014–2015 (26.2.%, 126/480) are being described for the first time. *M*. *pneumoniae*-positive specimens were identified using real-time PCR, as described previously [11].

### P1 gene typing

Nested PCR-restriction fragment-length polymorphism analysis was used for P1 genotyping, and was performed as previously described [9] directly from DNA extracted from the PCR-positive specimens. The PCR products of type 2 specimens were sequenced to identify type 2 variants.

### MLVA typing

Multiplex PCR amplification-linked capillary electrophoresis of four loci (Mpn13, Mpn14, Mpn15, and Mpn16) was used for the amended MLVA genotyping, and was performed according to previously described [10] and the international guidelines [12].

### Detection of macrolide resistance

Detection of macrolide resistance mutations, including the common macrolide resistance point mutations at positions 2063, 2064, 2611, and 2617 (*M*. *pneumoniae* numbering), was performed as previously described [13].

## Results and Discussion

### P1 gene typing

**Fig 1:**
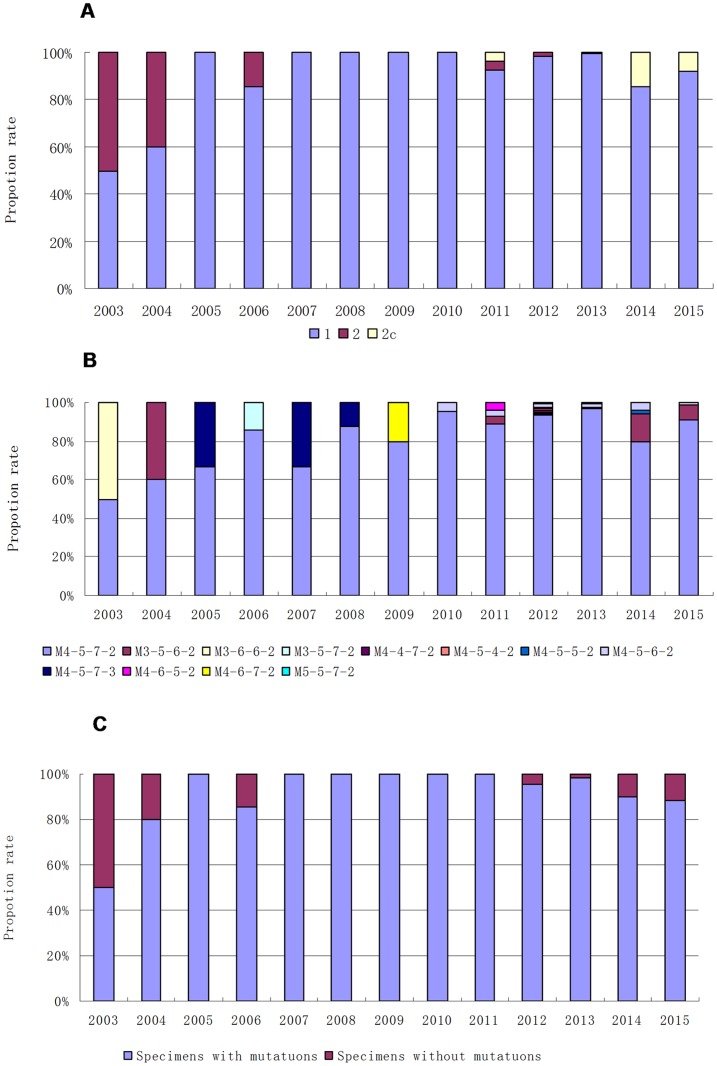
Prevalence rates of the different genotypes and macrolide resistance mutations by year. (A) Prevalence of the different P1genotypes from 2003–2015. (B) Prevalence of the different MLVA genotypes from 2003–2015. (C) Prevalence of macrolide resistance mutations from 2003–2015.

Twenty-three specimens were collected and analyzed between 2003 and 2007. Of these, 82.6% (19/23) contained *M*. *pneumoniae* identified as P1 type 1, while 17.4% (4/23) specimens were P1 type 2. Between 2014 and 2015, 126 specimens were analyzed, 89.7% (113/126) of which showed the presence of *M*. *pneumoniae* P1 type 1, while 10.3%, (13/126) were P1 type 2c. When combined with the previously published data from specimens collected from 2008–2013, among the 480 *M*. *pneumoniae*-positive specimens, 95.4% (458/480) were P1 type 1, 3.1% (15/480) were P1 type 2c specimens, and 1.5% (7/480) were P1 type 2 specimens (Table 1, Fig 1A). As 2007 and 2012 were epidemic years in China [3], the specimens were also grouped into three time periods. In the 5-year period from January 2003 until December 2007, types 2 and 2c accounted for 17.4% (4/23) of specimens, while between 2008 and 2013, after the epidemic of 2007, the prevalence of type 2 and 2c specimens significantly decreased to only 1.51% (5/331) (p = 0.001). However, from 2014–2015, after the epidemic of 2012, the percentage of type 2 and 2c specimens significantly increased (χ^2^ = 16.45, p<0.001) to 10.3% (13/126). While the P1-type genetic lineages varied slightly, there was no clear type-shift pattern similar to that observed by Kenri et al., in which type 2 strains were predominant from 1995–2001 in Japan, while type 1 strains were prevalent from 2002–2005 [8].

**Table 1:** Genotyping and macrolide resistance mutation results for *Mycoplasma pneumoniae* in clinical specimens collected from children in Beijing between 2003 and 2015.

Year	MLVA typing	P1 typing	Mutation	Number of specimens	Reference
2003	3-6-6-2	2	-	1	This study
2003	4-5-7-2	1	A2063G	1	This study
2004	3-5-6-2	2	A2063G	2	This study
2004	4-5-7-2	1	A2063G	2	This study
2004	4-5-7-2	1	-	1	This study
2005	4-5-7-2	1	A2063G	2	This study
2005	4-5-7-3	1	A2063G	1	This study
2006	3-5-7-2	2	A2064G	1	This study
2006	4-5-7-2	1	A2063G	5	This study
2006	4-5-7-2	1	-	1	This study
2007	4-5-7-2	1	A2063G	4	This study
2007	4-5-7-3	1	A2063G	2	This study
2008	4-5-7-2	1	A2063G	7	Xue et al [6]
2008	4-5-7-3	1	A2063G	1	Xue et al [6]
2009	4-5-7-2	1	A2063G	4	Xue et al [6]
2009	4-6-7-2	1	A2063G	1	Xue et al, Sun et al [6,9]
2010	4-5-6-2	1	A2063G	1	Xue et al, Sun et al [6,9]
2010	4-5-7-2	1	A2063G	21	Xue et al, Sun et al [6,9]
2011	3-5-6-2	2c	A2063G	1	Xue et al, Sun et al [6,9]
2011	4-5-6-2	1	A2063G	1	Xue et al, Sun et al [6,9]
2011	4-5-7-2	1	A2063G	23	Xue et al, Sun et al [6,9]
2011	4-5-7-2	2	A2063G	1	Xue et al, Sun et al [6,9]
2011	4-6-5-2	1	A2063G	1	Xue et al, Sun et al [6,9]
2012	3-5-6-2	2	A2063G	1	Xue et al, Sun et al [6,9]
2012	3-5-7-2	2	A2063G	1	Xue et al, Sun et al [6,9]
2012	4-4-7-2	1	A2063G	1	Xue et al, Sun et al [6,9]
2012	4-5-4-2	1	-	1	Xue et al, Sun et al [6,9]
2012	4-5-5-2	1	A2063G	1	Xue et al, Sun et al [6,9]
2012	4-5-6-2	1	-	2	Xue et al, Sun et al [6,9]
2012	4-5-6-2	1	A2063G	1	Xue et al, Sun et al [6,9]
2012	4-5-7-2	1	-	3	Xue et al, Sun et al [6,9]
2012	4-5-7-2	1	A2063G	130	Xue et al, Sun et al [6,9]
2012	4-5-7-3	1	A2063T	1	Xue et al, Sun et al [6,9]
2013	3-5-6-2	2c	A2063G	1	Yan et al [7]
2013	4-5-6-2	1	A2063G	2	Yan et al [7]
2013	4-5-7-2	1	-	2	Yan et al [7]
2013	4-5-7-2	1	A2063G	121	Yan et al [7]
2013	5-5-7-2	1	A2063G	1	Yan et al [7]
2014	3-5-6-2	2c	A2063G	2	This study
2014	3-5-6-2	2c	A2064G	1	This study
2014	3-5-6-2	2c	-	4	This study
2014	4-5-5-2	1	A2063G	1	This study
2014	4-5-6-2	1	A2063G	2	This study
2014	4-5-7-2	1	A2063G	37	This study
2014	4-5-7-2	1	G2611T,T2613C	1	This study
2014	4-5-7-2	1	-	1	This study
2015	3-5-6-2	2c	-	5	This study
2015	3-5-6-2	2c	A2063G	1	This study
2015	4-5-6-2	1	A2063G	1	This study
2015	4-5-7-2	1	-	4	This study
2015	4-5-7-2	1	A2063G	66	This study

−, No mutation was found.

### MLVA typing

Of the 23 specimens collected from 2003–2007, 16 were *M*. *pneumoniae* type M4-5-7-2, three were M4-5-7-3, two were M3-5-6-2, and the final two were M3-6-6-2 and M2-5-7-2, respectively. Of the 126 specimens collected from 2014–2015, 109 were *M*. *pneumoniae* type M4-5-7-2, 13 were M3-5-6-2, three were M4-5-6-2, and one was M4-5-5-2. When combined with the previously published specimens from 2008–2013, *M*. *pneumoniae* DNA corresponding to 12 distinct MLVA types was identified from among the 480 specimens (Table 1, Fig 1B). The most common type was M4-5-7-2 (91.0%, 437/480), followed by M3-5-6-2 (3.75%, 18/480) and M4-5-6-2 (2.08%, 10/480). Between the periods 2003–2007 and 2008–2013, the prevalence of type M3-5-6-2 gradually decreased when compared with the dominant MLVA type, M4-5-7-2 (p = 0.025). However, between 2008–2013 and 2014–2015, the prevalence of M3-5-6-2 gradually increased compared with M4-5-7-2 (χ^2^ = 20.8, p<0.001). These findings indicated a slight type change trend in the MLVA types M3-5-6-2 and M4-5-7-2.

In addition, new MLVA types were observed in every epidemic period. M3-6-6-2 and M4-6-7-2 were first detected in 2003 and 2009, respectively, while M4-4-7-2, M4-5-4-2, M4-5-5-2, M4-6-5-2, M5-5-7-2 and M4-5-6-2 were reported between 2010 and 2015, during and after the last worldwide epidemics [14, 15]. A possible correlation was observed between MLVA locus 13 and P1 typing: 99.8% of M4-5-7-2 specimens (436/437) were P1 type 1, and 100% of M3-5-7-2 (2/2), M3-5-6-2 (18/18), and M3-6-6-2 (1/1) specimens were P1 type 2 or 2c (Table 2).

**Table 2:** Relationship between MLVA type, P1 type, and macrolide resistance between 2003 and 2015.

MLVA type	Number of specimens	P1 type			Macrolide resistance	
		1	2	2c	Sensitive	Resistant
3562	18	0	3(16.7%)	15(83.3%)	9(50.0%)	9(50.0%)
3572	2	0	2(100%)	0	0	2(100%)
3662	1	0	1(100%)	0	1(100%)	0
4472	1	1(100%)	0	0	0	1(100%)
4542	1	1(100%)	0	0	1(100%)	0
4552	2	2(100%)	0	0	0	2(100%)
4562	10	10(100%)	0	0	2(20.0%)	8(80.0%)
4572	437	436(99.8%)	1(0.2%)	0	12(2.80%)	425(92.3%)
4573	5	5(100%)	0	0	0	5(100%)
4652	1	1(100%)	0	0	0	1(100%)
4672	1	1(100%)	0	0	0	1(100%)
5572	1	1(100%)	0	0	0	1(100%)

### Detection of macrolide resistance mutations

Of the 23 specimens from 2003–2007, 87% (20/23) were positive for macrolide resistance mutations in the 23S rRNA gene of *M*. *pneumoniae*, while 88.9% (112/126) of the 126 specimens from 2014–2015, contained macrolide resistance mutations. When combined with the previously published specimens from 2008–2013, 94.8% (455/480) of the 480 *M*. *pneumoniae*-positive specimens contained macrolide resistance mutations in the 23S rRNA gene and, of these, 99.3% (452/455) had an A2063G mutation. The remaining three specimens contained A2064G (n = 2) and A2063T (n = 1) mutations.

As the *M*. *pneumoniae* macrolide resistance rates vary from 2.0–13.2% in most European countries and the United States [16], and are up to 90.0% in China and Japan [15,17], this type of antibiotic resistance has become a focus of worldwide research. In this study, the rates of mutation varied throughout the 13-year study period (Fig 1C). Analysis of data from the three different time periods showed that the resistance mutation rates rose from 87% (20/23) in the period 2003–2007 to 97.6% (323/331) in the period 2008–2013 (p = 0.028), and then dropped to 88.9% (112/126) in 2014–2015 (χ^2^ = 15.05, p<0.001). This trend of an increase followed by a decline in macrolide resistance rates echoes that of the changes in P1 genotype prevalence.

**Fig 2:**
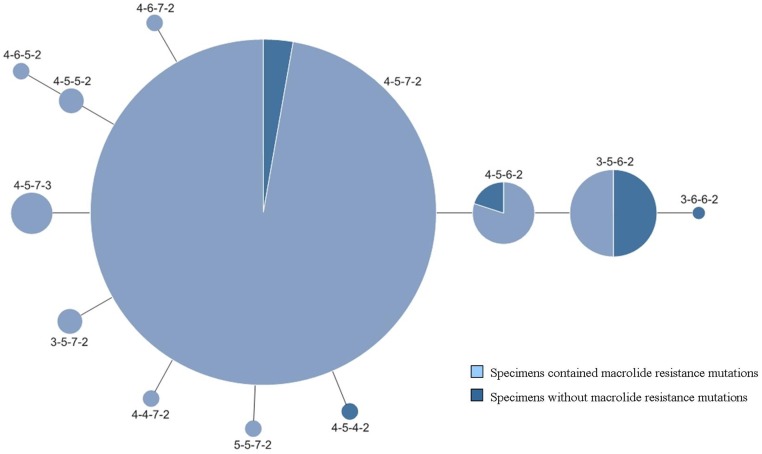
Relationship between MLVA type and the presence of macrolide resistance mutations from 2003–2015.

A report by Qu et al. [18] showed a possible association between MLVA type M3-5-6-2 and macrolide susceptibility, and our previous findings [9] showed a possible association between M4-5-7-2 and macrolide resistance. In this study, the macrolide resistance mutations were distributed across 10 distinct MLVA types. Among them, 93.4% were M4-5-7-2 (425/455) and 1.98% were M3-5-6-2 (9/455). Of the 25 specimens with no detectable macrolide resistance mutations, one specimen contained *M*. *pneumoniae* DNA that was grouped into genotype M3-6-6-2 (1/25, 4%), one was M4-5-4-2 (1/25, 4%), nine were M3-5-6-2 (9/25, 36%), two were M4-5-6-2 (2/25, 8%), and eight were M4-5-7-2 (8/25, 32%) (Table 2, Fig 2). While 50% (9/18) of M3-5-6-2-type specimens had no macrolide resistance mutations, 97.3% (425/437) of M4-5-7-2-type specimens contained resistance mutations (Table 2, Fig 2). In addition, rates of resistance mutations increased along with the increase of the M4-5-7-2 type between 2003–2007 and 2008–2013, and decreased along with the decrease of the M4-5-7-2 type during 2014 and 2015. These findings further indicated that there may be an association between the four-locus MLVA genotypes and macrolide resistance.

This study examined changes in the molecular characteristics of *M*. *pneumoniae* in clinical specimens collected from children in Beijing over a 13-year period. The genotype M4-5-7-2/P1 was most prevalent in children with *M*. *pneumoniae* infection, especially from 2008 to 2013. However, the prevalence of the two main MLVA types, M4-5-7-2 and M3-5-6-2, along with the main P1 gene types, 1 and 2, varied between the three epidemic periods. In particular, some genotypes were only detected in Beijing during and after the last worldwide epidemics. Our study also provides more evidence that there is an association between MLVA type M4-5-7-2 and macrolide resistance.

However, this study also has some limitations. As the number of specimens from 2003–2007 was small, examination of a greater number of specimens will help to better understand the epidemic trends. Moreover, other typing methods, such as pulsed-field gel electrophoresis and multilocus sequence typing, could be used in conjunction with the current methods to enhance the results of further studies [19,20]. Although a large amount of sequencing is required with multilocus sequence typing, this method has better discriminatory power than MLVA and P1-restriction fragment-length polymorphism analysis. Therefore, more studies using a combination of these methods should be undertaken to investigate associations between clinical infections and specific *M*. *pneumoniae* molecular characteristics.
